# Investigation on the Microporosity Formation of IN718 Alloy during Laser Cladding Based on Cellular Automaton

**DOI:** 10.3390/ma14040837

**Published:** 2021-02-09

**Authors:** Hao Lv, Zhijie Li, Xudong Li, Kun Yang, Fei Li, Hualong Xie

**Affiliations:** 1 Department of Mechanical Engineering and Automation, Northeastern University, Shenyang 110819, China; lvhao@me.neu.edu.cn (H.L.); 1800309@stu.neu.edu.cn (Z.L.); 1800308@stu.neu.edu.cn (X.L.); 2 School of Mechanical Engineering, Southeast University, Nanjing 211189, China; yk1873639934@163.com; 3 Department of Information Science and Engineering, Shenyang University of Technology, Shenyang 110870, China; lifei@sut.edu.cn

**Keywords:** Inconel 718 alloy, cellular automaton, square algorithm, micro-porosity, laser cladding

## Abstract

Porosity is one of the most common defects in the laser cladding of Inconel 718 (IN718) alloy, which can reduce the strength and fatigue performance of the components. However, the dynamic formation of microporosity is challenging to observe through experiments directly. In order to explore the formation mechanism of porosities and dynamically reproduce the competitive growth between porosities and dendrite, a multi-scale numerical model was adopted, combined with a cellular automaton (CA) and finite element method (FEM). The decentered square algorithm was adopted to eliminate crystallographic anisotropy and simulate dendrite growth in different orientations. Afterward, based on the formation mechanism of microporosity during solidification, equiaxed and columnar dendrites with porosities were simulated, respectively. Dendrite morphology, porosity morphology, and distribution of solute concentration were obtained during the solidification process. The simulation results were reasonably compared with experimental data. The simulation results of the equiaxed crystal region are close to the experimental data, but the columnar crystal region has a relative error. Finally, the interaction effects of porosities and dendrites under different environmental conditions were discussed. The results suggested that with the increase in the cooling rate, the quantity of porosity nucleation increased and the porosity decreased.

## 1. Introduction

**Figure 1:**
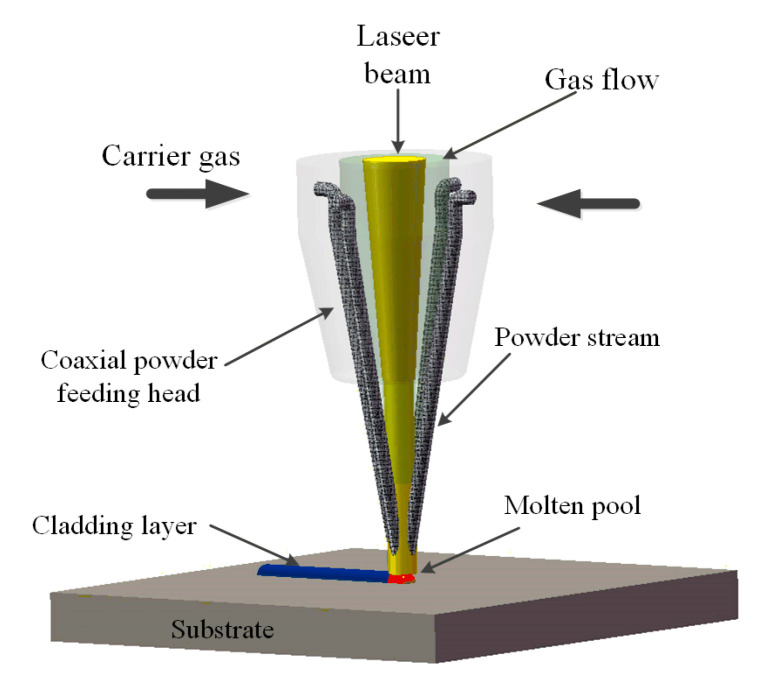
Laser cladding process schematic.

Laser cladding (Figure 1) is a kind of additive manufacturing technology, which uses a laser beam to rapidly heat and melt the alloy powder and the surface of the substrate. After rapid solidification, a surface coating with a low dilution rate and a metallurgical bond is formed [1]. Laser cladding can directly fabricate parts with dense structure, uniform composition, excellent performance [2,3]. In addition, it can be applied in the field of repair components [4,5,6]. Therefore, it has been involved in many industrial areas.

IN718 alloy has excellent mechanical properties, high tensile strength at 700 °C and high oxidation resistance at 1000 °C. It has stable chemical properties at low temperatures and is applied in various parts of jet engines and gas turbines, as well as power plants [7]. Porosities are a common defect in additive manufacturing and can cause internal damage and cracking, thereby reducing the fatigue life of the material. Porosities are considered to be the cause of crack initiation and failure and stress concentration under loading conditions [8]. The fatigue life of specimens can be predicted according to the size and location of porosity [9]. The microporous defects of alloys during solidification can be divided into two typical types: shrinkage porosity and gas porosity [10,11]. The former is mainly due to insufficient liquid feeding during the shrinkage and solidification of the alloy, while the latter is caused by the insoluble to discharge hydrogen gas holes during solidification. It has been proven that shrinkage porosity is harder to form than gas porosity during solidification. The molten metal inevitably contains certain gas components. Due to the different solubilities of the gas in the solid and liquid phases, it will precipitate the supersaturated gas in the liquid phase to form microscopic porosities as the solidification progresses. Hydrogen has a high solubility in liquid IN718 alloy, and when the temperature drops below the freezing point of the IN718 alloy, the solubility of hydrogen will decrease sharply [12,13]. In the process of cooling and solidification of the molten pool, along with temperature decreasing dramatically, hydrogen exceeding the solubility limit will escape from the solid phase. If the hydrogen cannot escape from the molten pool before it is solidified, hydrogen porosities will eventually form. Hydrogen porosity is a major defect in the laser cladding process and has been reported by many researchers. Durandet et al. [14] investigated how to minimize the formation of porosity in the laser cladding. Yang et al. [15] observed two types of hydrogen porosities in laser cladding: one is chain-shaped porosities generated along the bottom of the molten pool, and the other is larger porosities with a diameter greater than 100 μm dispersedly distributed in the coating. By changing the laser power and powder feed rate, larger holes can be eliminated entirely. Pei et al. [16] found that the porosity defects increased with laser beam velocity in AlSi40 FGM laser cladding. Zhang et al. [17] applied an electric-magnetic compound field to change the stress state of porosities in the molten pool, which obviously influenced the overflow speed of porosities. In summary, experiments and simulations have been used to understand the mechanism of porosity defects during solidification. The laser cladding process has the characteristics of rapid cold and rapid heat, and the opacity of metal materials largely limits the real-time observation of the evolution between porosities and dendrites. The experiment is mainly X-ray and post-solidification observation during the solidification process [14,15,16,17]. In addition, the melt pool complex physical, including hydrodynamics and heat dissipation phenomenon, has not been fully understood. Peiyu et al. [18] found that the final position of porosities was determined by the combination of Marangoni flow, powder–gas-flow impacts, and boundary motion. Ng et al. [19] proposed that gas bubbles would be retained by Marangoni-driven flow and made a reasonable explanation that the melt pool geometry would tend to dominate the flow direction of the gas bubbles. Panwisawas et al. [20] suggested that morphological evolution of porosity during additive manufacturing (AM) is caused by the change of flow pattern in the melt pool, which was dictated by forces including the Marangoni force and recoil pressure. V.G. et al. [21] developed a hydrodynamic model based on the open computational fluid dynamics package OpenFoam, which took the heat dissipation geometry into account. However, it is inconvenient to understand the nucleation mechanism and evolution law of porosities during solidification. Based on this, the simulation of porosity formation and evolution has become a test and method to control the porosity formation in the solidification process.

Researchers have established various predictive models for predicting the formation and evolution of microporosity. The predictive models are mainly divided into the following categories: criterion function model based on the empirical formula [22], intergranular flow model [23], hydrogen diffusion control porosity growth model, and diffusion control-stochastic model [24,25,26,27]. At present, the nucleation process used to describe the microporosity is mainly based on a heterogeneous nucleation model, combined with corresponding empirical criteria. Many scholars have carried out the microstructure evolution during alloy solidification based on different prediction models. In particular, cellular automata (CA) and phase field (PF) are the most commonly used computational models to simulate the evolution of solidification microstructure among various models [28,29,30,31,32,33,34,35,36]. Lee et al. [28] and Dong et al. [29] established a dendrite and porosity growth coupling model by using CA to simulate the interaction between grain structure and porosity at a microscopic scale. Zhu et al. [30] used the CA method to simulate the formation of micro porosities, dendrite growth and hydrogen concentration evolution during the solidification of Al-7wt%Si alloy. Gu [31] simulated the mutual development of microscopic porosities and dendrites during welding. Wu [32,33], Sun [34], etc., coupled the multi-phase flow lattice Boltzmann (LB) model with the CA approach to simulate dendrite growth, porosity nucleation and growth, and movement in 2D and 3D regions. Du [35] adopted PF method to simulate columnar dendrites and porosities. However, the effect of multiple equiaxed dendrites and porosities is not considered. PF method can effectively couple multiple physical fields without tracing complex interfaces in the simulation process. On the contrary, PF method has a large amount of calculation and low efficiency, and it has certain limitations in the simulation of microstructure evolution of the molten pool. The cellular automata method is based not only on the physical mechanism of grain nucleation and growth but also on a ‘probabilistic’ random idea, which can consider the randomness in the evolution of the organization.

In this paper, the CA-FE model was used to simulate the microstructure growth and porosity formation of IN718 alloy during solidification. By adopting the cellular automata method, the simulation of the grain growth process with different preferred orientation angles is completed. Equiaxed and columnar dendrites with porosities were simulated, respectively. The direct objective of this work is to simulate the formation and growth of hydrogen porosities in laser cladding and to explore the influence of different solidification conditions on the porosity distribution. In addition, this paper provides a complete attempt and understanding of the formation of porosities during laser cladding. It is believed that this study is useful for research aimed at improving the performance of IN718 coatings by controlling porosities in laser cladding.

## 2. Model Description and Numerical Algorithm

### 2.1. Macro-Scale Transient Temperature Field Simulation by the Finite Element Model (FEM) Model

The laser cladding process is simulated and a single-track finite element model of laser cladding is established. Laser cladding is a typical transient thermal analysis. Dendrite growth and porosity evolution of the temperature field is derived from the heat conduction equation

(1) $$\frac{\partial }{\partial x}\left(\lambda \frac{\partial T}{\partial x}\right)+\frac{\partial }{\partial y}\left(\lambda \frac{\partial T}{\partial y}\right)+\frac{\partial }{\partial z}\left(\lambda \frac{\partial T}{\partial Z}\right)+Q=\rho c\frac{\partial T}{\partial t}$$

where *λ* is the thermal conductivity, *T* (*x*, *y*, *z*, *t*) is the transient temperature, *Q* is the input capacity, *ρ* is the density of the material, *c* is the specific heat, *t* is the direct interaction time. In the simulation, various thermophysical parameters of the cladding layer and substrate are set as a function of temperature.

In the process of laser cladding, the material will undergo solid and solid–liquid phase changes, which will affect the accuracy of finite element simulation. It is difficult to describe the latent heat of phase change directly, which is deal with by defining the enthalpy characteristics of the material, and the change of enthalpy can be described as a function of density, specific heat and temperature. The relationship between them is shown in Formula (2).

(2) ΔH=∫ρC(T)dT

A double ellipsoid heat source is used to simulate the movement of the laser heat source. The temperature field distribution of laser cladding is that the temperature gradient in the region before the heat source center is enormous. In contrast, the temperature gradient distribution in the latter half of the ellipsoid is slow. Goldak [36] proposed a double ellipsoid power density distribution heat source model. The heat generation rate distribution rate of a point inside the ellipsoid of the front hemisphere along the *x*-axis is

(3) $$q\left(x,y,z\right)=\frac{6\sqrt{3}f_{1}\eta UI}{aba_{1}\pi^{\frac{3}{2}}}\exp(\frac{-3x^{2}}{a_{1}{}^{2}})\exp(\frac{-3y^{2}}{b^{2}})\exp(\frac{-3z^{2}}{c^{2}})$$

The heat generation rate distribution rate of a point in the ellipsoid of the rear hemisphere along the *x*-axis is

(4) $$q\left(x,y,z\right)=\frac{6\sqrt{3}f_{2}\eta UI}{aba_{2}\pi^{3/2}}\exp(\frac{-3x^{2}}{a_{2}{}^{2}})\exp(\frac{-3y^{2}}{b^{2}})\exp(\frac{-3z^{2}}{c^{2}})$$

where *q* is heat generation rate, *a*_1_, *a*_2_, *b*, *c* are the shape parameters of the double ellipsoid heat source, *η* is the thermal laser efficiency, *U* is the voltage, *I* is the current, *f_1_* and *f_2_* are the energy distribution coefficients of the front and back half of the heat source model, respectively, satisfying *f*1 + *f*2 = 2.

**Figure 2:**
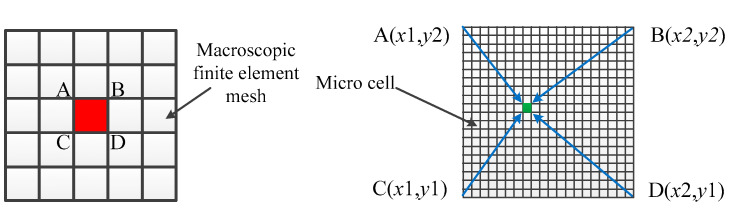
Macro-micro coupling diagram.

The evolution of the macroscopic temperature field and microstructure requires the coupling of the heat transfer model and dendrite growth model. In order to simulate the solidification conditions under different process parameters, the weakly coupled method is adopted. For a small region of the cladding layer, the temperature values calculated in the finite elements are used to carry out the micro-simulation. The node temperature in the finite element is derived and a polynomial is fitted. The temperature values of each cell at different times are obtained by bilinear interpolation, as shown in Figure 2. The corresponding temperature is provided for the simulation of the microstructure under the molten pool.

**Figure 3:**
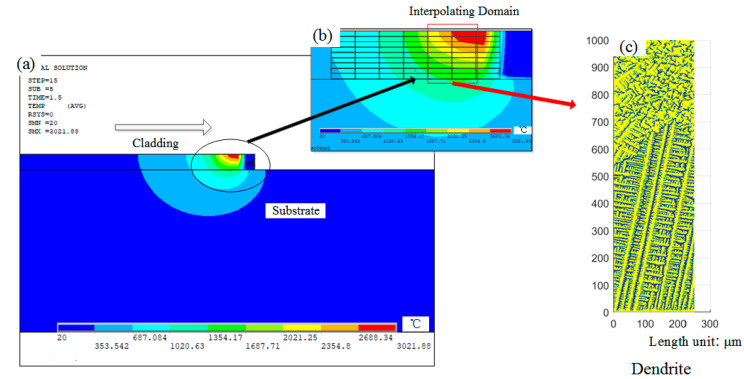
FEM simulation result of laser cladding, (**a**) temperature distribution, (**b**) the whole interpolating domain [38], (**c**) results of microstructure simulation.

The size of the FEM model substrate is 35 × 20 × 10 mm (length × width × height). The contour of the cladding layer is a specified curve that fits according to experimental results [37]. Since the structure and load of the FEM model are symmetric, the analysis is carried out by taking half and applying symmetric constraints on the symmetric plane. In addition to the melting width is 2.4 mm and the melting height is 1 mm. The simulation process parameters are shown in Table 1 based on a previous experiment [38]. The configuration of the FEM model is described in Figure 3, which is adapted from reference [38]. Gradient meshing is adopted to ensure the fine mesh at the cladding layer and the matrix mesh size far away from the cladding layer gradually increases, which can improve the computational efficiency [39]. By analyzing the calculated results, the isothermal surface of the temperature field shows an ellipsoidal distribution, and the simulation results have been proved by literature [38]. The range of the molten pool is 0.5 × 1 mm, and the finite element mesh size is 0.25 × 0.1 mm, which is divided into microcells of 1 × 1 μm to simulate dendrite evolution. A columnar-to-equiaxed transition (CET) criterion is developed to describe the formation and reproduce the grain size and morphologies, as shown in Figure 3c, which proves that the model of temperature field and dendrite growth transformation model is correct and provides an idea to choose different regions for the coupling of dendrites and porosities [40,41]. Besides, the properties of the IN718 alloy required for the simulation are listed in Table 2.

### 2.2. Mathematical Model of Dendrite Growth

The mathematical model of microstructure evolution and the performance of Inconel 718 adopted are mainly based on the method in literature [43]. Besides, Kothe et al. [44] proposed a way to calculate the average local interface curvature, which can reflect the change of interface shape more accurately. The basic equation is

(5) $$\bar{k}=\frac{2\frac{\partial fs}{\partial x}\frac{\partial fs}{\partial y}\frac{\partial^{2}fs}{\partial x\partial y}-{\left(\frac{\partial fs}{\partial x}\right)}^{2}\frac{\partial^{2}fs}{\partial y^{2}}-{\left(\frac{\partial fs}{\partial y}\right)}^{2}\frac{\partial^{2}fs}{\partial x^{2}}}{{\left[{\left(\frac{\partial fs}{\partial x}\right)}^{2}+{\left(\frac{\partial fs}{\partial y}\right)}^{2}\right]}^{3/2}}$$

In the simulation, the randomness of grain growth direction is considered. Therefore, the decentered square algorithm is adopted to simulate the dendrite growth to satisfy any angle. The decentered square algorithm was first proposed by Gandin and Rappaz [45]. After improved by Wang et al. [41,46], it is an ideal method to simulate multi-angle dendrite growth at present.

**Figure 4:**
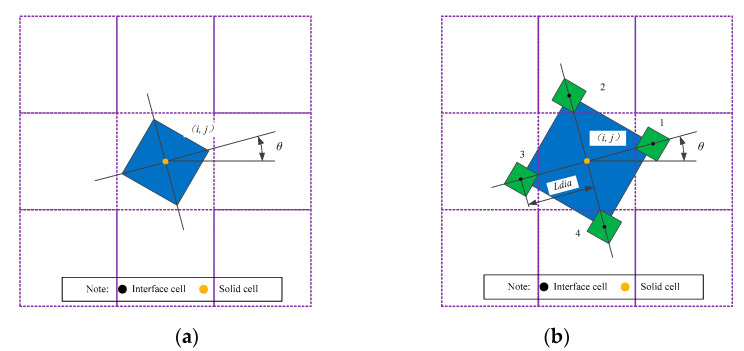
Decentered square algorithm (**a**) nucleus grows with square envelope (**b**) captured new interface cells have specific crystallographic orientations and envelope centers [41].

Figure 4 shows the decentered square algorithm to describe symmetric dendrite growth. When the nucleation condition is satisfied in the liquid phase, nucleation occurs at cells (*i, j*) and *θ* is a random angle, as shown in Figure 4a. A virtual square was generated at the center of the cell (*i, j*), and the diagonal of the square was the optimal growth direction during the solidification process. Driven by solute diffusion and dendrite growth kinetics, the solid fraction keeps increasing. *Ldia* changes with the solid phase fraction of the cell (*i, j*) until the solid phase fraction reaches the maximum. During the expansion process, the coordinate values of the four vertices of the virtual square produced are based on the following formula

(6) $$\begin{array}{c} y_{1}=Ldia*cos\theta +y_{0}\\ x_{1}=Ldia*sin\theta +x_{0}\\ y_{2}=-Ldia*sin\theta +y_{0}\\ x_{2}=Ldia*cos\theta +x_{0}\\ y_{3}=-Ldia*cos\theta +y_{0}\\ x_{3}=-Ldia*sin\theta +x_{0}\\ y_{4}=Ldia*sin\theta +y_{0}\\ x_{4}=-Ldia*cos\theta +x_{0} \end{array}$$

where *x*_0_, *y*_0_ are the coordinate values of the cell (*i, j*), *x*_1_–*x*_4_ and *y*_1_–*y*_4_ are the coordinates of the four vertices corresponding to the decentered square, *θ* is a random angle, *Ldia* is the maximum length of the half diagonal of the decentered square.

When the four vertices of the square touch the surrounding cells, the touched cell is captured as an interface cell, and *θ* is assigned to the captured cell as well. The new nucleus positions of these captured cells are located at the four vertices of the square in Figure 4b. Then the cell grows and captures in the same way. In order to ensure that the decentered square can pass through the cell along the growth direction and reduce its redundant capture as much as possible, the maximum length of the half diagonal of the square is taken as [47].

(7) $$Ldia=\frac{1.414*fs}{\max\left(cos\theta ,sin\theta \right)}$$

where *fs* is the solid fraction of the cell (*i, j*).

**Figure 5:**
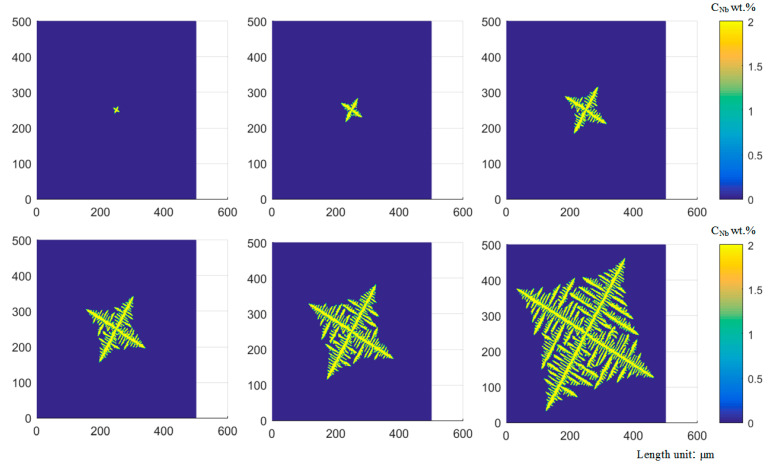
The growth and evolution process of a single dendrite when the growth direction is 60°.

Figure 5 describes the growth process of a single dendrite with a growth direction of 60°. When the nucleation condition is satisfied, a nucleation core is formed at the center of the cell, and then the core begins to grow in the optimal growth direction. In the initial stage of solidification, the solid–liquid interface is relatively stable, and the dendrite does not have apparent secondary dendrite arms, and the dendrites mainly grow in the morphology of the primary dendrite arms. As the solidification progresses, the solute concentration in the front of the dendrite interface gradually increases, and a more developed secondary dendrite arm will be produced in the front of the primary dendrite arm. Due to the higher solute content between the primary dendrite arms, the growth rate of the secondary dendrite arms is lower. It can be seen from Figure 5 that the distribution of dendrites is symmetrical. Besides, the growth and distribution of primary and secondary dendrites are more consistent with the actual situation.

### 2.3. Analysis of Dendrite Growth Simulation Results

**Figure 6:**
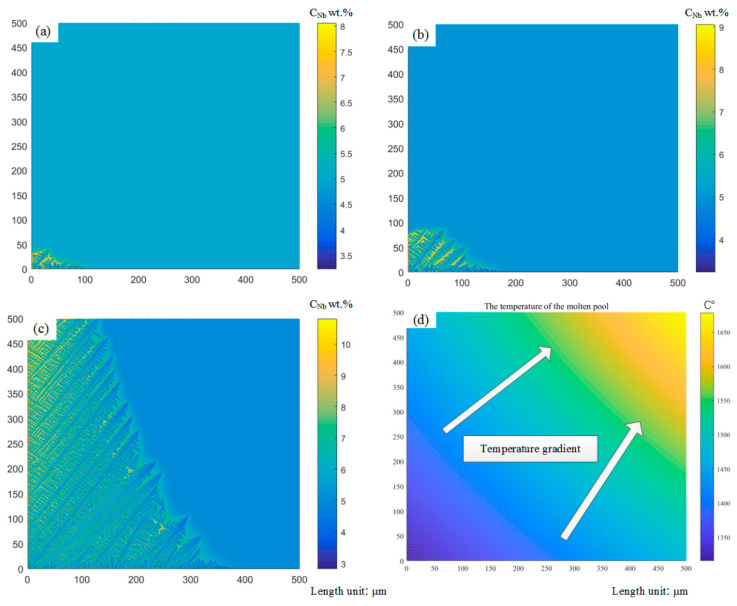
Simulated evolution of columnar grain structure, (**a**) dendrites nucleation, (**b**,**c**) dendrites growth, (**d**) the temperature of the molten pool. Domain size is 500 × 500 μm.

The dendrite morphology in the laser cladding layer mainly includes equiaxed dendrites and columnar dendrites. The competitive growth of columnar crystals was simulated for different preferred orientation angles of dendrites, as shown in Figure 6. It can be demonstrated from Figure 6a that at the beginning of solidification, the grains have just begun to nucleate and grow in all directions. At this time, the effect of the preferred direction is small. The dendrites with different preferred orientations have shown individual differences. The growth of columnar dendrites whose preferred orientation is inconsistent with the direction of the temperature gradient has slightly lagged behind the columnar dendrites whose preferred orientation is consistent with the direction of the temperature gradient. When the solidification process proceeds to Figure 6b, it can be found that some columnar dendrites have been blocked. It is worth noting that the preferred orientation of the blocked columnar dendrites is closer to the temperature gradient direction than other adjacent columnar dendrites. It is blocked because the solute discharged to the surrounding region during the growth of columnar dendrites with the preferred orientation consistent with the temperature gradient increases the solute concentration at the tip and decreases the growth rate. It can be seen from Figure 6c, the dendrites whose preferred orientation is inconsistent with the direction of the temperature gradient are blocked. On the contrary, the dendrites whose preferred orientation is consistent with the direction of the temperature gradient continue to grow. In addition, dendrites, whose secondary dendrite orientation is consistent with the temperature gradient direction, begin to grow significantly. Figure 6d of the temperature distribution during the operation was intercepted, and it was demonstrated that the direction of dendrite growth was roughly the same as the gradient direction, which verified the accuracy of the model.

**Figure 7:**
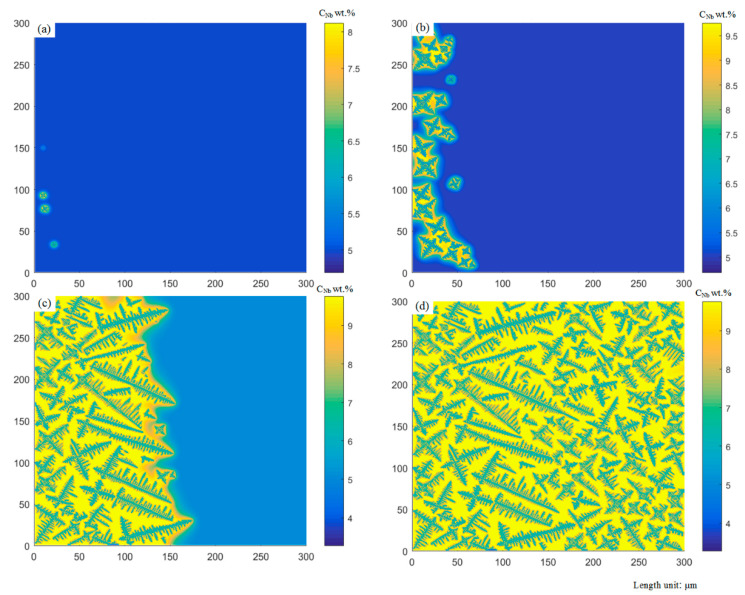
Simulated evolution of equiaxed grain structure, (**a**) dendrite nucleation, (**b**,**c**) dendrite growth, (**d**) end of equiaxed dendrite growth. Domain size is 300 × 300 μm.

Inside the molten pool, when the temperature gradient in the liquid phase is small, the liquid phase temperature curve and the crystallization temperature curve intersect at a long distance, forming a large component undercooled region in the liquid phase. Due to the large region of undercooled composition, new dendrite nuclei may be formed. The surrounding states of these dendrites nuclei are almost the same and grow into nearly symmetrical equiaxed crystals, as shown in Figure 7.

## 3. Mathematical Model of Porosity Evolution

It is demonstrated that the evolution of porosity is influenced by gas segregation and solidification shrinkage. For gas porosity, hydrogen gas porosities in IN718 alloy are considered in this paper. The solubility of hydrogen in liquid nickel is pretty evident, and the melt in the molten pool may absorb a large amount of hydrogen at high temperatures. On the contrary, the solubility of hydrogen in solid nickel is quite difficult, and the solubility decreases with the decrease of temperature. After the molten pool begins to solidify, the solubility of hydrogen changes abruptly. As the solidification process progresses, the growth of dendrites not only discharges solutes but also discharges hydrogen. The hydrogen concentration in the liquid phase increases and accumulates in the liquid phase at the front of the solid–liquid interface, which forces the nucleation of porosities heterogeneously [48].

### 3.1. Nucleation Model of Porosity

In this paper, the stochastic model proposed by Lee [49,50] to describe the nucleation of the porosity nucleation model is adopted. When the gas dissolved in the liquid ($C_{L}^{H}$) exceeds a critical supersaturation level, and porosity begins to nucleate. The essential criterion function of nucleation supersaturation of stomata is

(8) $$\frac{dn^{H}}{dV}=\{\begin{array}{l} \frac{N_{max}^{H}}{\left(S_{max}^{H}-S_{min}^{H}\right)};\;\;(C_{L}^{H}/S_{L}^{H}>S_{n}^{H})\\ 0;\;\;\;\;\;\;\;\left(C_{L}^{H}/S_{L}^{H}\leq S_{n}^{H}\right) \end{array}$$

where $dn^{H}/dV$ is the porosity distribution equation, $N_{max}^{H}$ is the maximum nucleation density, $S_{max}^{H}$ and $S_{min}^{H}$ are the maximum and minimum nucleation saturation, respectively, which can be measured indirectly through experiments. $C_{L}^{H}$ and $S_{L}^{H}$ are local hydrogen concentration and local hydrogen saturation respectively, $S_{n}^{H}$ is the critical saturation criterion of nucleation.

According to Sievert’s law, the solubility of hydrogen in IN718 alloy can be calculated by the formula [51]

(9) $$S_{L}^{H}=K^{H}\sqrt{\frac{P_{g}}{P_{ref}}}$$

where *P_g_* is the internal pressure of a gas porosity, $P_{ref}$ is the standard atmospheric pressure, $K^{H}$ is Sievert constant. In IN718, the Sieverts constant regarding hydrogen solubility can be calculated by the formula [52]

(10) $${\mathrm{lgK}}^{H}=\frac{-2577}{T}+3.014+\varphi$$

where *T* is temperature, φ is the influence of other alloying elements in the molten liquid on the solubility of hydrogen. In this paper, the impact of *φ* on *K* is ignored. The internal pressure of gas porosity, *P_g_*, only includes the effects of the atmospheric pressure and surface energy pressure, which can be written as

(11) $$P_{g}=P_{ref}+\frac{\lambda_{G}}{r_{G}}$$

where $\lambda_{G}$ is the surface tension of the gas–liquid interface, $r_{G}$ is the radius of porosity.

### 3.2. Growth of Microporosity

Assuming that the porosities are only affected by environmental pressure and surface tension, buoyancy and other external forces are not considered. The gas volume increasing of the porosities precipitated during the solidification process in a time step can be calculated by the formula

(12) $$\Delta V=N\left(H\right)\frac{RT}{Pg}$$

where *N(H)* is the amount of hydrogen absorbed by all interface cells of the porosity, *R* is the gas constant, and *T* is the local temperature. The hydrogen absorbed at all gas–liquid interfaces during porosity growth should be considered, which can be evaluated by

(13) $$N_{H}=\underset{x\in J}{\sum }\left(1-f_{g}^{\left(i,j\right)}-f_{s}^{\left(i,j\right)}\right)\left(C_{H}^{l}-S_{H}^{l}\right)V_{c}$$

where *fg* and *fs* are the gas fraction and solid phase fraction of the gas–liquid interface cells, respectively, *Vc* is the volume of a cell. The gas volume increment of the cell (*i, j*) at the gas–liquid interface can be calculated by the formula

(14) $$\Delta V\left(i,j\right)=\Delta V\frac{G_{g}\left(i,j\right)}{\sum G_{g}}$$

where *Gg (i, j)* is the geometrical factor of the gas–liquid interface cell (*i, j*), $\sum G_{g}$ is the sum of geometrical factors of all gas–liquid interface cells of the porosity. The geometrical factor *Gg* is related to the state of neighbor cells, which is defined by

(15) $$G=min\left[1,\frac{1}{2}\left(\overset{4}{\underset{m=1}{\sum }}s_{m}^{I}+\frac{1}{\sqrt{2}}\overset{4}{\underset{m=1}{\sum }}s_{m}^{II}\right)\right]\;,\;s^{I},s^{II}=\{\begin{array}{l} 0\;\;\;\;\;(f_{g}<1)\\ 1\;\;\;\;\;\left(f_{g}=1\right) \end{array}$$

where *s^Ⅰ^* and *s^Ⅱ^* is the state of the nearest neighbor cells and the second-nearest neighbor cells, respectively.

According to the volume increase of the porosity at each time step, the gas fraction increase of each gas–liquid interface cell can be calculated by the formula

(16) $$\Delta f_{g}^{\left(i,j\right)}=\frac{\Delta V_{}^{\left(i,j\right)}}{V_{c}}$$

The gas fraction varies over time. Similar to the dendrite model, the gas fraction of the cell (*i, j*) can be expressed as

(17) $${f_{g}|}_{i+1}={f_{g}|}_{i}+\Delta f_{g}$$

When *fg* (*i, j*) = 1 through cyclic calculation, the state of the cell changes from the gas–liquid interface to the gas phase. In the next cycle, the liquid cell surrounding the newly formed gas phase cell is captured as the gas–liquid interface cell to calculate the next step of stomatal growth.

### 3.3. Coupling Growth of Porosities and Dendrites

During the solidification process, the growth of the solid phase produces a concentration gradient of hydrogen at the solid–liquid interface, and the growth of porosities produces a concentration gradient of hydrogen at the gas–liquid interface, which redistributes the accumulated hydrogen. The concentration of the hydrogen discharged from the growth of the solid phase at the solid–liquid interface satisfies the equilibrium distribution condition:

(18) $$C_{s}^{*}H=k_{H}C_{l}^{*}\left(H\right)$$

where *k_H_* is the equilibrium partition coefficient, $C_{s}^{*}H$ is the equilibrium concentration of hydrogen in the solid phase at the solid–liquid interface, $C_{l}^{*}\left(H\right)$ is the equilibrium concentration of hydrogen in the liquid phase at the solid–liquid interface.

At the gas–liquid interface, if the local hydrogen concentration $C_{l}\left(H\right)$ is greater than the local hydrogen saturation $S_{l}\left(H\right)$, the supersaturated gas will be absorbed by the porosities. In this case, the reduction of hydrogen concentration at the gas–liquid interface needs to be considered. In the presence of porosities, the porosities will continue to absorb supersaturated hydrogen in the liquid phase at the gas–liquid interface, causing them to grow slowly. These two phenomena lead to differences in the hydrogen concentration in the liquid phase, and a hydrogen concentration gradient appears, which provides a driving force for hydrogen diffusion. The diffusion of hydrogen can also be described as

(19) $$\frac{\partial C_{L}^{H}}{\partial t}=\frac{\partial }{\partial x}\left[D_{L}^{H}\frac{\partial C_{L}^{H}}{\partial x}\right]+\frac{\partial }{\partial y}\left[D_{L}^{H}\frac{\partial C_{L}^{H}}{\partial y}\right]+\frac{\partial }{\partial z}\left[D_{L}^{H}\frac{\partial C_{L}^{H}}{\partial z}\right]-\left(1-f_{s}-f_{G}\right)\left(C_{l}^{H}-S_{l}^{H}\right)V_{CA}$$

During the solidification of the laser cladding molten pool, the growth of dendrites and the evolution of porosities influence each other.

By using Equations (8)–(19), the porosity nucleation, solute segregation and diffusion, interaction of porosities and dendrites, etc., during solidification can be calculated in one step time followed by another calculation circle until the state of all cells in the interpolating domain becomes solid. The calculation method for the steady-state time step of solute diffusion is

(20) $$\Delta t^{H}=\frac{1}{4.5}\min\left(\frac{\Delta x^{2}}{D_{L}^{H}},\frac{1}{\Delta f_{g.max}^{}}\right)$$

where Δx is the size of a cell, *Dl(H)* is the maximum value of the diffusion coefficient of H in the liquid phase when the zone temperature is the highest. $\Delta f_{g.max}^{}$ is the maximum growth rate of the solid phase fraction per time step, including the maximum growth rate of the solid phase fraction of the two phases during the eutectic reaction.

## 4. Experimental Results and Discussion

**Figure 8:**
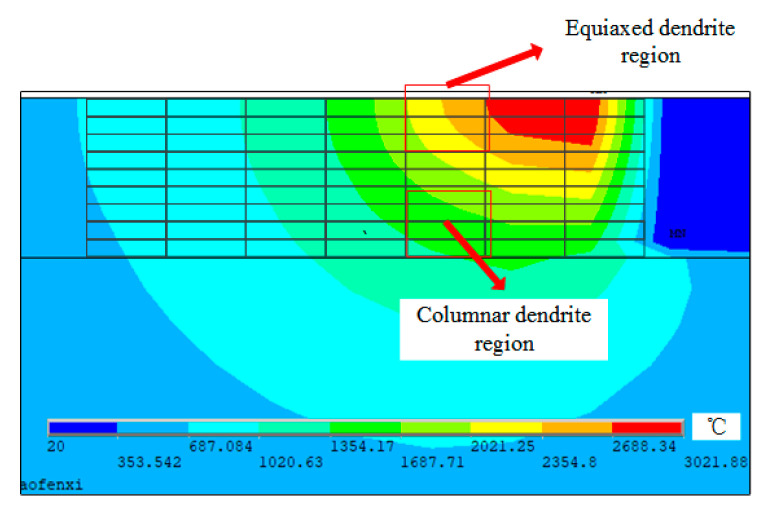
Typical equiaxed and columnar region of the observation.

The experiment was executed by laser cladding of IN718 alloy with the model simulation, and the substrate size is 100 × 100 × 8 mm. The metallographic samples were prepared, and the cladding layer was run through by wire cutting equipment along the scanning direction. The surface of the substrate is polished off with sandpaper, then washed with absolute ethanol and acetone in turn, and dried. The cross-sections of cladding are etched by Calling reagent (40 mL HCl, 40 mL ethanol, 2 g CuCl_2_). The porosity information given in this research is the percentage of porosity statistics in the cladding layer, the observation region of which is obtained as shown in Figure 8, respectively. For the equiaxed dendrite and columnar dendrite regions, simulations and experiments were carried out for multiple measurement comparisons. The simulation results are mainly measured based on the software MATLAB. For the experimental data, software Image is used to perform binarization and calculate the porosity and the quantity of porosities.

### 4.1. Evolution of Microstructure during Solidification

**Figure 9:**
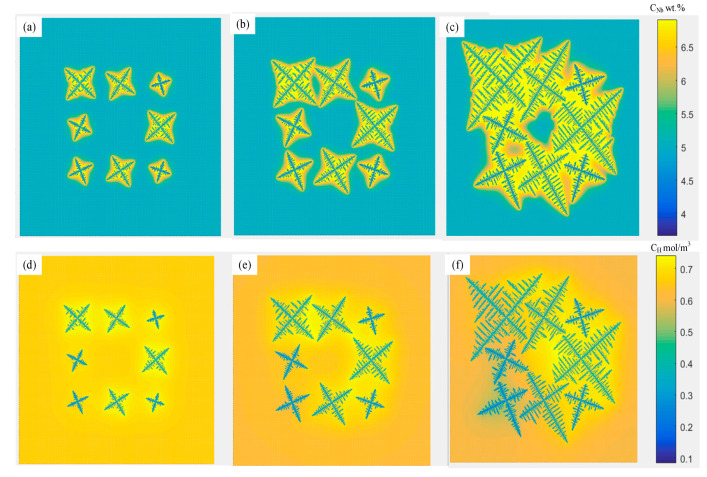
The morphology of dendrites in the liquid phase was shown by solute concentration (**a**–**c**) and H concentration (**d**–**f**), respectively.

The established porosity and dendrite coupling model is performed to simulate the growth of multiple dendrites in IN718 alloy, as shown in Figure 9. The calculation region is divided into 300 × 300 μm uniform square grids with a mesh size of 1 μm. Several dendrites nuclei are randomly set in the calculation region in advance. The solute concentration and hydrogen concentration are used to show the concentration changes in the solid and liquid cells, respectively. The morphology of dendrites and the evolution of the concentration field are shown in Figure 9. From Figure 9a,d, it can be seen that in the initial stage of solidification, the individual dendrites are far apart and grow independently. When the two dendrites are relatively close to each other, the two dendrites on the upper left in Figure 9b,e touched earlier. The competition of dendrite growth occurs, mainly caused by the changes in the concentration field around the dendrites. In Figure 9c,f, the dendrite arms are already well developed, and the secondary dendrite arms have grown significantly. It turns out that the solute distribution is reasonable.

**Figure 10:**
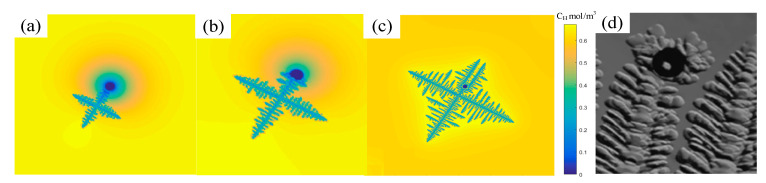
Morphology evolution of the dendritic microstructures with a fixed volume porosity. (**a**–**c**) evolution process (**d**) experimental result [32].

The solidification process with fixed-volume porosity was investigated. The effect of bubbles on the formation of dendrites is shown in Figure 10. First, a porosity is placed in the liquid phase to form a nucleus. As time goes by, dendrites and porosities meet the growth conditions and grow continuously. When the tip of the dendrite encounters a bubble, the tip of the dendrite will be blocked by the bubble and split into two tips, and the other tips will grow at a normal speed. Due to the interaction between bubbles and dendrites, the bubbles are also deformed. These simulation results are consistent with the experimental results of the bubble interface microstructure [32], as shown in Figure 10d.

### 4.2. Dendrites and Multi-Bubble Interaction Evolution

According to the established coupling model of dendrite growth and porosities evolution, the constructed random angle orientation rule, the equiaxed dendrites growth and porosities evolution are simulated. According to the nucleation time, the initial time is set to 2.037 s, and the initial hydrogen concentration is set to 0.6 mol/m^3^. When the temperature gradient in the liquid phase is sufficiently small, equiaxed dendrites will form inside the molten pool.

**Figure 11:**
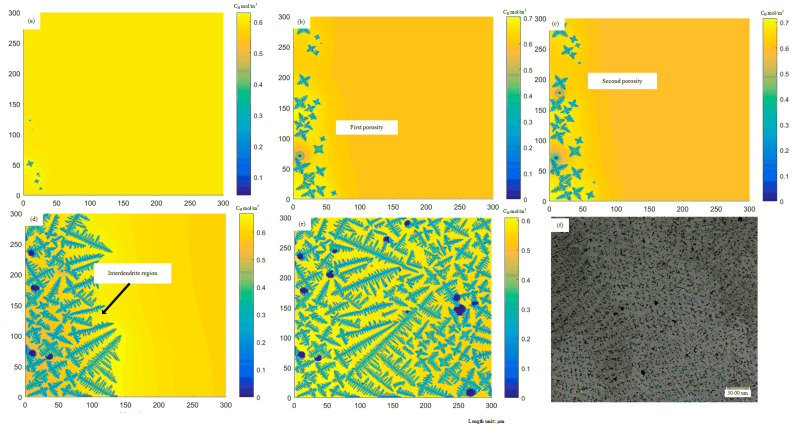
Simulated evolution of equiaxed dendrites growth with porosities in the molten pool at different times: (**a**) t = 2.0390 s, (**b**) t = 2.0419 s, (**c**) t = 2.0452 s, (**d**) t = 2.0725 s, (**e**) t = 2.1012 s. (**f**) is observed from the test result. Domain size is 300 × 300 μm.

It can be seen from Figure 11 that, as the temperature decreases, when t = 2.0390 s, the equiaxed dendrites have nucleated and formed protrusions in their preferred growth direction. According to the moving direction of the laser beam in the laser cladding process, the entire molten pool is nucleated from left to right and from top to bottom. Due to the nucleation and growth of equiaxed crystals, the hydrogen concentration distribution is affected by the hydrogen discharged from the front of the solid–liquid interface. The hydrogen concentration near the equiaxed dendrite is relatively high. When the hydrogen concentration in the liquid phase rises to higher than the supersaturation of porosity nucleation, the porosity begins to nucleate. When t = 2.0419 s, the first porosity meets the nucleation condition and approximates spherical nucleation, as shown in Figure 11b. When t = 2.0452 s, the second porosity has completed nucleation near the equiaxed dendrites and the nucleated porosity continues to expand. As the solidification progresses, the primary dendrites of different equiaxed dendrites coarsen and grow further. Different equiaxed dendrites start to contact each other and hinder each others’ growth, and the formation of porosities also destroys the growth of dendrites. When t = 2.0725 s, the shape of the grains is elongated along the grain boundary related to the heat source distribution of the double ellipsoid. Finally, when t = 2.1012 s, several relatively complete equiaxed crystal morphologies can be seen in the simulation region. After the solidification is completed, if the formed porosities are hindered by dendrites and cannot leave the liquid phase, porosities will eventually be formed. The porosities become irregular due to the morphology of the surrounding dendrites. Comparing the experimental observation results in Figure 11f, the porosity model can simulate the micro porosities and dendrite morphologies extraordinarily similar to the experimental results.

According to the nucleation time, the initial time is set to 2 s. The grow direction angle is set to 60°, in order to ensure consistency with the experimental angle. At the edge and middle of the laser cladding molten pool, the structure of dendrites is mainly columnar dendrites.

**Figure 12:**
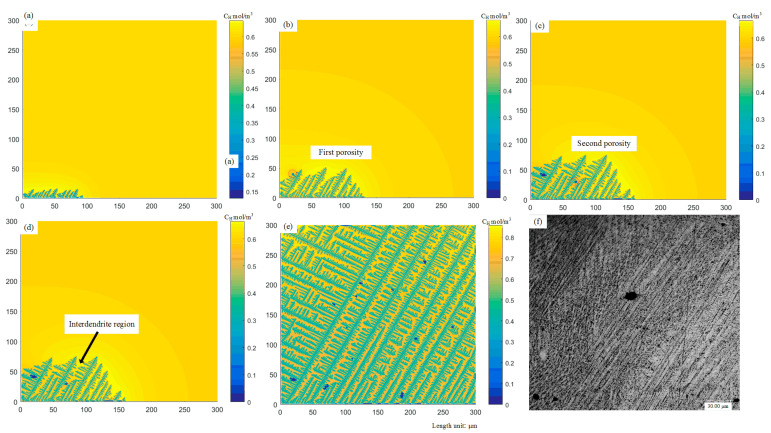
Simulated evolution of columnar dendrites growth with porosities in the molten pool at different times: (**a**) t = 2.0105 s, (**b**) t = 2.0112 s, (**c**) t = 2.0120 s, (**d**) t = 2.0160 s, (**e**) t = 2.0210 s. (**f**) is observed from the test result. Domain size is 300 × 300 μm.

It can be seen from Figure 12 that when t = 2.01 s, as the temperature decreases, the dendrites begin to nucleate and grow along the oblique direction. When t = 2.0112 s, the nucleation of porosity occurred, and the dendrites gradually approached and hindered each other. When it continued to grow to t = 2.0160 s, more porosities nucleation appeared, and the porosities grew significantly. It can be noticed that when the bubble grows large enough, the growth of dendrites can be blocked entirely. When it grows to t = 2.0210 s, the proportion of porosities in the simulated region also increases significantly. In addition, comparing the experimental observation results in Figure 12f, the porosity model also can simulate the microscopic porosities and dendrite morphologies similar to the experimental results.

**Figure 13:**
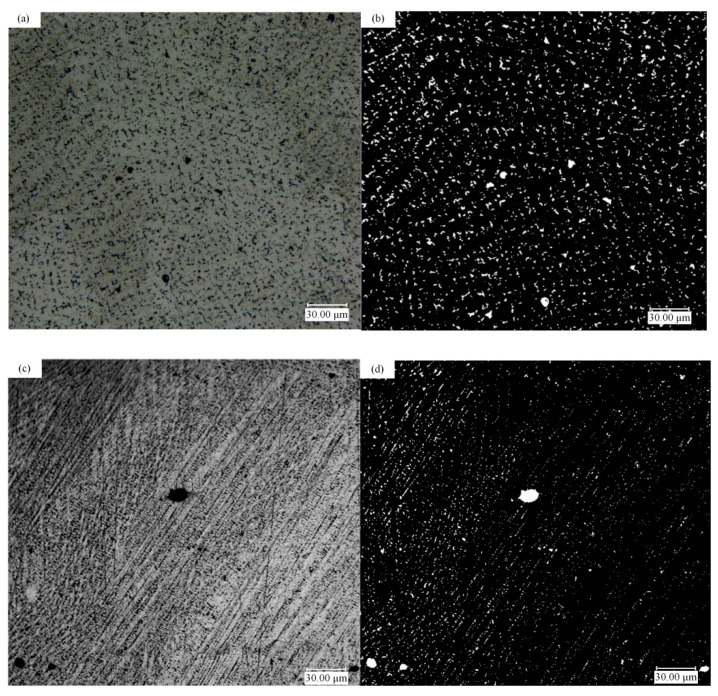
Schematic diagram of experiment data processing: (**a**,**b**) equiaxed and binary porosity treatment; (**c**,**d**) columnar and porosity binary treatment.

The experimental data intercepted the position corresponding to the simulation region. For the equiaxed crystal regions, simulations and experiments were carried out for multiple measurement comparisons. Because the nucleation of the porosity is also random, the experimental image and simulation do not fit so well. In terms of simulation, under the same process parameters, the formation and angle of each dendrite conform to randomness, which also ensures that the formation of porosity is uncertain. Five times of experimental sample preparation and analysis were carried out. A clear porosities distribution map is obtained by polishing. Afterward, it is etched to ensure the distribution of dendrites (Figure 13a,c) and used by software Image to perform binarization (Figure 13b,d). Finally, the results are listed in Table 3. According to Table 3, for porosity, the average porosity of experimental results is 1.381%, and the simulation data is 1.574%. The standard deviation of the former is 0.91%, and the standard deviation of the corresponding model is 1.58%. For the quantity of porosities, the average value of the experimental results is 21.8, and the average value of the simulation is 22. The above two points verify the accuracy of the simulation in equiaxed crystal regions.

The comparison between the simulation results and the experimental data is presented for the columnar crystal region. The average porosity is 2.3%, and the average quantity of porosities is 15 in the model results, the error of which is about 31%. In the upper left corner of Figure 12e, since dendrites grow freely in the CA model, there are cubic dendrites and a small quantity of quaternary dendrites, which are not consistent with reality. In order to ensure the randomness of the model, no mandatory transformation is implemented in its position, so there will be some deviation from the actual part. It may be due to the large gap between Figure 12f and the actual process. Based on the above situation, in the next section, the simulation of the cooling conditions on the porosities is explored mainly in the equiaxed crystal region.

### 4.3. Influence of Solidification Conditions on the Morphology of Porosities

Growth and microstructure of porosities evolution under different substrate temperatures are simulated. The quantitative relationship between the different substrate temperature and temperature gradient can be expressed by the formula [53]

(21) $$G=\frac{2\pi K{\left(T-T_{0}\right)}^{2}}{\eta P}$$

where *G* is the temperature gradient between the substrate and the cladding layer, *T* is the liquidus temperature of the IN718 alloy, *T*_0_ is the preheating temperature of the substrate, η is the laser absorption rate, *P* and *K* are the laser power and the thermal conductivity of the material, respectively. Hunt et al. [54] researched and proposed the columnar-to-equiaxed transition model, changing different cooling methods to affect the dendrite distribution and dendrite segregation [55,56,57], which affect the porosities. It is of great benefit to understand the formation and distribution of porosities under different solidification conditions. The cooling conditions are shown in Table 4.

**Figure 14:**
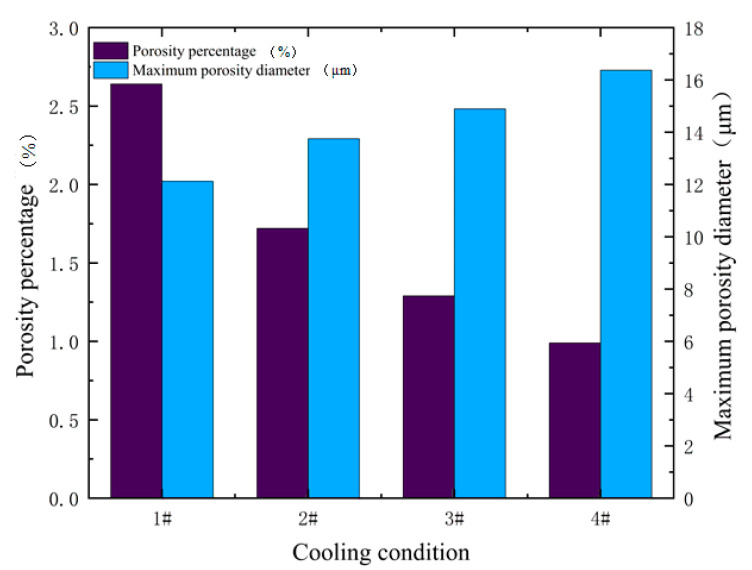
Results of porosity and maximum porosity diameter under different cooling conditions.

Figure 14 shows the changes in the quantity of porosity and maximum porosity diameter under different cooling conditions. It can be seen from the simulation results that as the cooling rate increases, the porosity of the IN718 alloy increases. On the contrary, the maximum size of the porosities decreases, the results of which have a similar trend with the previous study [31]. The influence of cooling conditions on the morphology of porosities is reflected in two aspects. On the one hand, according to the relationship between dendrite spacing and cooling rate: $d=80\lambda^{-0.33}$, where *d* is dendritic space and λ is cooling rate [42]. It can be demonstrated that the dendritic space decreases with respect to the increase in the cooling rate. The smaller the dendrite arm spacing is, the more limited the diffusion range of the molten pool is. If the cooling rate is fast, the dendrite structure will form earlier and hinder the diffusion of H in the liquid phase, which limits the size of the porosities. On the other hand, different cooling rates will affect the time of H diffusion and porosities growth in the liquid phase. As the cooling rate decreases, there is enough time for H to diffuse, which will cause more H to diffuse to the front of the porosities. As the cooling rate decreases, there is enough time for H to diffuse, which will cause more H to diffuse to the front of the porosities. When the growth time of the porosity gets longer, the diameter of the porosity increases, which is consistent with the trend of the study [30].

**Figure 15:**
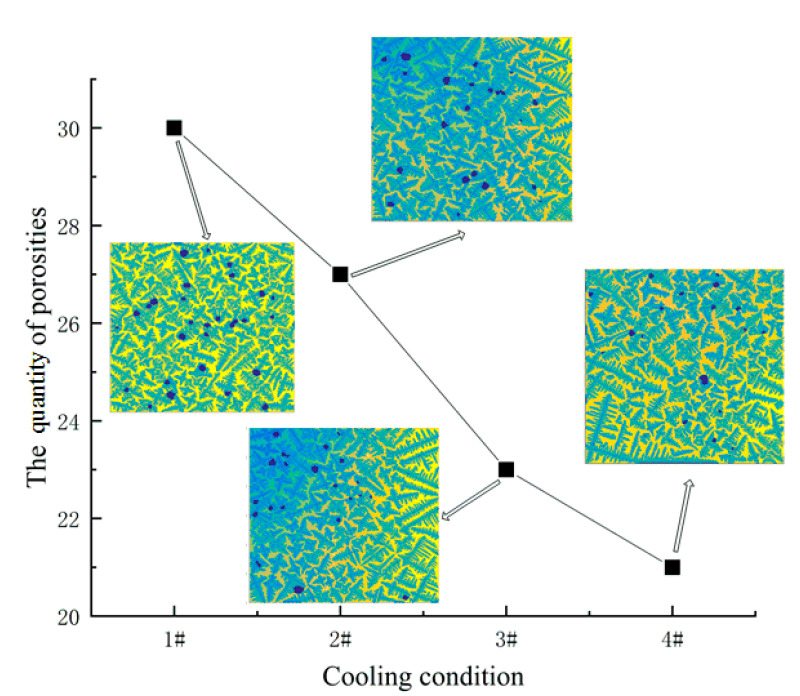
Results of the quantity of porosities under different cooling conditions.

From Figure 15, it is indicated that as the cooling rate increases, the porosities distribution becomes more uniform and the shape is more regular. The greater the cooling rate, the greater the quantity of dendrite nuclei. As the growth temperature decreases rapidly, the time for porosity growth is significantly shortened. At the same time, there will be a competitive growth phenomenon between porosities and dendrites. As a result, there is a phenomenon that the quantity of porosities is increasing but the size of which is shrinking.

## 5. Conclusions

In this paper, a multi-scale numerical model is adopted to simulate the dynamic formation of dendrites during the laser cladding process and porosities, which includes porosity nucleation, dendrite growth, solute redistribution, and diffusion of hydrogen. In addition, dendrite evolution and porosity formation under different cooling conditions are visually simulated. The conclusions obtained are as follows:

1. CA-FEM is adopted to simulate the microstructure of laser cladding, which predicts the competitive grain growth under the dendrite tip of behavior and interaction between grain growth kinetics. According to the total supercooling degree, the grain cells can grow faster in the preferential growth direction, which can reflect the growth rate of the grain cell and the growth behavior of the granule. Only when the preferred orientation of dendrite growth is consistent with the direction of the maximum temperature gradient can it continue to grow. The preferential growth direction of dendrite also has an essential influence on the competition process between dendrites.
2. The established model for the coupled growth of dendrites and porosities comprehensively considers the nucleation and growth of dendrites and porosities in the liquid phase, solute redistribution and diffusion and interface curvature effects. It can demonstrate the microstructure morphology of the coupled growth of dendrites and porosities in good agreement with the experimental observations. It indicates that the porosities are affected by the growth of dendrites, and the presence of porosities will also hinder the growth of dendrite.
3. The simulation results were reasonably compared with experimental data in different typical regions. In the equiaxed region, for porosity, the average porosity of experimental results is 1.381%, and the simulation data is 1.574%. For the quantity of porosities, the average value of the experimental results is 21.8, and the average value of the simulation is 22. In the columnar region, it has a relatively large error.
4. The competitive growth of dendrites and porosities was simulated, and the influence of different cooling conditions on the formation of porosities was analyzed. It is concluded that as the cooling rate increases, the quantity of porosities is increasing, and the porosity increases, while the maximum of the porosities is decreasing. The model can be applied to optimize the additive manufacturing process in order to obtain the desired results.

## Figures and Tables

**Table 1:** Process parameters of the simulation.

Parameters	Laser Power (W)	Scanning Speed (mm/s)	Powder Federate (g·min^−1^)	Shield Gas Flow (L·min^−1^)
-	1500	10	18	15

**Table 2:** Properties of IN718 alloy parameters in CA model.

Parameter	Symbol	Units	Value	Ref.
Liquidus temperature	T*_l_*	K	1678	[42]
Eutectic temperature	T*_eu_*	K	1643	[42]
Slope of the liquidus line	m*_L_*	K/(mass)%	−10.5	[42]
Diffusion coefficient of Nb in liquid	D*_L_*(Nb)	m^2^/s	3 × 10^−9^	[38]
Diffusion coefficient of Nb in solid	D*_S_*(Nb)	m^2^/s	1 × 10^−12^	[38]
Diffusion coefficient of H in liquid	D*_H_*(Nb)	m^2^/s	2.8 × 10^−7^	[12]
Diffusion coefficient of H in solid	D*_H_*(Nb)	m^2^/s	6.7 × 10^−10^	[12]
The partition coefficient of Nb	k_0_	/	0.48	[42]
The partition coefficient of H	k_H_	/	0.069	[25]
Maximum pore nucleation saturation	$S_{\max}^{H}$	/	2.0	[25]
Minimum pore nucleation saturation	$S_{\min}^{H}$	/	1.4	[25]
Timestep of Nb	t	s	0.6 × 10^−4^	/
Timestep of H	t	s	0.1 × 10^−5^	/

**Table 3:** Comparison between experiment and model about porosity and the quantity of porosity.

Sample Number	1#	2#	3#	4#	5#
Porosity of experiment (%)	1.08	0.335	1.14	1.535	2.813
The quantity of porosities in the experiment	26	25	20	15	23
Porosity of model (%)	1.51	1.31	1.59	1.51	1.95
The quantity of porosities in the model	22	21	20	23	24

**Table 4:** Different cooling conditions.

Cooling Condition	Liquid Nitrogen Cooling [55]	The Ice Water [56]	At Room Temperature	Preheating [57]
Boundary Temperature/°C	−196 (1#)	0 (2#)	23 (3#)	400 (4#)

## Data Availability

Data sharing not applicable.
